# The application of nanotechnology in treatment of Alzheimer’s disease

**DOI:** 10.3389/fbioe.2022.1042986

**Published:** 2022-11-17

**Authors:** Yanyan Cao, Run Zhang

**Affiliations:** Department of Neurology, First Affiliated Hospital of Wannan Medical College, Wuhu, China

**Keywords:** nanotechnology, nanomedicine, brain disorder, Alzheimer, chronic disease

## Abstract

The buildup of beta-amyloid plaques in the brain results in Alzheimer’s disease (AD), a neurodegenerative condition. A permanent treatment for AD is not yet available. Only a slowing down of its advancement is possible with the current pharmaceutical options. Nevertheless, nanotechnology has proven to be advantageous in medical applications. It has a lot of potential for AD therapy, particularly in diagnosing the condition and providing an alternative course of treatment. In this review, we outline the developments and benefits of nanomedicines in treating AD. Prospective nanomedicines for diagnosing and surveillance therapeutic interventions for AD and other diseases of the central nervous system (CNS) may be clinically accessible, persuading the development of investigation in this field.

## Introduction

The most prevalent type of dementia, Alzheimer’s disease (AD), affects close to 50 million people worldwide. Because of the rise in the average life expectancy, it is anticipated that this population will reach 150 million in the year 2050 (Gaudreault and Mousseau, 2019). Then, AD is expected to remain a clinical, social, and economic concern. Several approaches are being investigated to find novel treatments for AD (Loureiro et al., 2014). Dealing with the pathology of AD has some restrictions. The intranasal administration of drugs targeting neurotransmitters or enzyme modulation is exploited to treat the cognitive deficits caused by AD (Sood et al., 2014). Only four treatments for AD have been approved by the Food and Drug Administration (FDA), and these all target different aspects of the disease’s two main molecular pathways: the buildup of Aβ peptide and neurofibrillary tangles (NFT) of p-tau protein (Sabbagh, 2020). However, using these medications causes a significant increase in therapy failures because of their poor absorption in the neuronal cell membranes, instability, neurotoxicity, and a number of other pharmacokinetic and pharmacodynamic characteristics (Suri et al., 2015; Guo et al., 2020). This emphasizes the necessity for developing alternative therapeutic interventions. Recognition of different molecular targets that could result in new treatments is anticipated to result from the finding of new biomarkers, which are also expected to strengthen previous AD diagnoses. The development of novel therapies needs to describe the pathophysiological mechanisms underlying AD and the best biomarkers to discover them. Furthermore, it is essential to deliver diagnostic and therapeutic compounds to the target sites in these mechanisms effectively and precisely. Because of their diverse chemical properties and their predisposition for chemical change to modulate and refine desired characteristics, nanoparticles (NPs) have enabled significant advancements in drug delivery, treatment, and disease diagnosis (Duskey et al., 2017; Mulvihill et al., 2020). Due to the enhancement of the medicine’s pharmacological effects, the necessary doses to generate therapeutic effects are reduced when drugs are delivered using nanocarriers. This results in a reduction in the number of adverse effects experienced by patients (Wais et al., 2016). The main components of NPs involve a wide range of substances encapsulating compounds with various chemical properties, including lipids, polymers, and metals. The ability of NPs to deliver compounds to difficult-to-reach organs, like the CNS, where the blood-brain barrier (BBB) must be crossed and controlled kinetic drug releases are necessary for long-term therapies, is indispensable for chronic CNS diseases (Wilczewska et al., 2012). The purpose of this study is to provide a high-level summary of how AD treatment has evolved as a direct result of the application of nanotechnology. A strong focus is also put on the significant challenges that currently exist as well as the prospects for the future of this industry.

## Alzheimer’s disease pathogenesis and molecular basis

**FIGURE 1:**
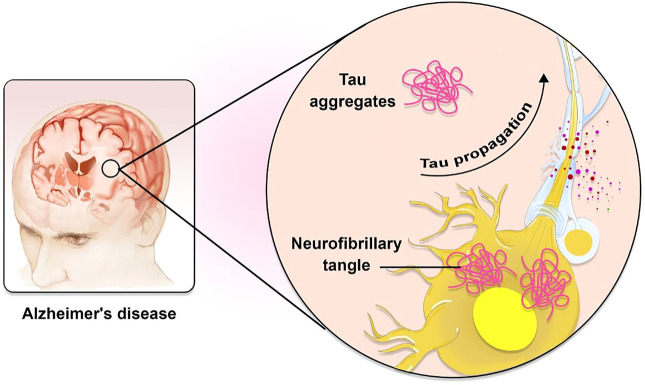
The role of Tau in AD pathogenesis. Tau is known to spread in a predictable way along neuronal networks in neurofibrillary tangles (NFTs). It is possible that the characteristic development of AD neuropathology is caused by interneuron transfer of the pathogenic form of tau.

AD is a massively complicated and progressive neurodegenerative disorder (Association, 2015). Extracellular accumulations of Aβ plaques and intracellular accumulations of NFTs constituted of hyperphosphorylated microtubule-associated τ have been reported as AD’s histopathological features. Plaques composed of Aβ first appear in the brain’s basal, temporal, and orbitofrontal neocortices. As the disease progresses, however, it spreads throughout the neocortex, hippocampus, amygdala, diencephalon, and basal ganglia. In severe cases, the mesencephalon, cerebellar cortex, and lower brain stem contain Aβ. The development of tau-tangles, which can be observed in the locus coeruleus and the transentorhinal and entorhinal regions of the brain, is triggered when a concentration of Aβ is present. It propagates to the hippocampus and neocortex during the critical phase (Figure 1) (Goedert, 2015). Also, AD development is influenced by several physiological variables summarized in Table 1.

**TABLE 1:** The involvement of physiological factors in AD pathogenesis.

Variable	Distinction	Reference
Hypertension	It is thought that hypertension can influence AD through its relationship with cerebrovascular pathology. Hypertension, commonly known as high blood pressure, is another factor that may have a role in the pathophysiology of AD. According to the research, hypertension is the cause of increased plaque development in the brain	Nehls (2016)
Homocysteine	It contributes to the progression of beta amyloid (Aβ) plaque development. In addition, there is speculation that homocysteine plays a role in the elevation of oxidative stress in the brain, which is a factor in the advancement of AD diseases	Pacheco-Quinto et al. (2006)
Inflammation	A significant correlation with the overall risk of dementia	Mrak and Griffin (2005)
Physical activity	Exercising produces anti-inflammatory effects and offers a myriad of additional advantages through a variety of routes, all of which have the potential to slow or stop the advancement of AD.	Schlegel et al. (2019); Valenzuela et al. (2020)
Diabetes mellitus	There is a theoretical support has been depicted on the relationship between diabetes and Alzheimer’s disease, along with novel strategies to prevent diabetic people from developing AD. It has also been found that AD can be alleviated by taking preventative measures against diabetes such as maintaining a healthy weight and diet, participating in sports, and engaging in other physically active pursuits	Sun et al. (2020)

Despite the progress made in understanding the biology of the AD and developing treatments for it, we do not yet have a molecule that may postpone and/or prevent the progression of the disease in aged people and patients with AD. There are two distinct etiologies of AD: early-onset familial AD and late-onset sporadic AD. Investigations of Alzheimer’s disease brains obtained *via* autopsies found that there are no discernible differences between the early-onset familial form of the disease and the late-onset sporadic form (Ayodele et al., 2021). Early-onset familial AD is caused by mutations in three different loci: the amyloid precursor protein (APP), presenilin 1 (PS1), and presenilin 2 (PS2). It is hypothesized that these genetic changes make a person more susceptible to increased mitochondrial dysfunction (Hyman et al., 2012; Reddy et al., 2018). If a person has these genetic antecedents, it is more probable that they will get AD in their 50s rather than in their 70s or later in life. In addition, having the apolipoprotein E allele 4 (APOE4) increases a person’s risk of developing late-onset sporadic AD since this allele plays a role in the accumulation of amyloid protein (Hyman et al., 2012). In addition, the development of both types of AD is caused by age-related variables such as reactive oxygen species (ROS), malfunction in mitochondria, and phosphorylation of tau (Kritsilis et al., 2018; Reddy et al., 2018). Additionally, synaptic loss and mitochondrial dysfunction are early events or triggers in the progression of the disease. Mitochondria, sometimes known as the “powerhouses of the cell,” are accountable for the vast majority of the metabolic activities that are necessary for human survival. Unfortunately, when these super organelles are not functioning properly, it leaves us open to the development of serious, debilitating illnesses (Oliver and Reddy, 2019).

## Blood-brain barrier in Alzheimer’s disease

The BBB plays a pivotal role in the movement of biomolecules into and out of the brain’s neuronal system. Hence, to enhance the delivery of drugs to the brain, it is necessary to gain a comprehension of the structural and functional features of the BBB (Sharifzad et al., 2019; Alahmari, 2021). Two specialized barriers, the cerebrospinal fluid barrier (CSFB) and BBB distinguish the complex human CNS (Pathan et al., 2009; Dabbagh et al., 2022). Both cognitive impairment and dementia are caused by cerebrovascular dysfunction, which may result in cerebral amyloid angiopathy in AD. In addition, it acts as a mediator for the buildup of Aβ peptides within the brain. The advanced glycation end product (RAGE) receptor and the low-density lipoprotein receptor related protein 1 (LRP1) are the two main receptors that enable the BBB to control the regulation of Aβ transport to the brain (Sagare et al., 2012).

The perseverance required for the selective transport of small molecules over the BBB is provided by cohesive domains bound to the endothelial cells (Abbott et al., 2010). A regulated intracellular transport *via* transcytosis takes place to fulfill the needs of proteins and peptides for brain homeostasis. Endothelial cells, with the assistance of a wide variety of specialized transporting proteins, can enable the transport of molecules. However, this ability is contingent on the nature of the compounds (hydrophilic or hydrophobic). In preclinical investigations, several various nanocarriers have been reported as having the potential to cure brain illnesses like AD. To cross the BBB, these carriers encapsulate the AD medications as cargo (Mulvihill et al., 2020).

## Nanotechnology in Alzheimer’s disease

Nanotechnology aims to design, produce, and use nanomaterials-materials with at least one dimension falling between 1 and 100 nm. At this scale, materials frequently exhibit bulk-independent properties that are considered interesting to the medical community, including superparamagnetism or surface plasmon resonance (Khan et al., 2019). Additionally, because proteins and nucleic acids are in the same size range as nanomaterials, particularly nanoparticles, they are well-suited for interacting with those biomolecules and, as a result, with cells. Likewise, a large surface-volume ratio associated with nanometric size offers benefits in applications of biological recognition, particularly in sensing. Therapeutic uses of these compounds have undergone extensive testing (Auría-Soro et al., 2019; Li et al., 2019; He et al., 2021). Applications of nanomaterials have also been studied in the field of precision medicine for the past few years (Mura and Couvreur, 2012). Because of the drug’s inability to circumvent the BBB, the only treatment for AD currently on the market focuses on symptomatic relief. Due to its many benefits, nanotechnology-based therapy may overcome this restriction (Ling et al., 2021). The FDA has given its blessing for the use of commercially available medications to a wide variety of nanocarriers that range in size from very tiny to very large. These nanocarriers are used in the treatment of neurological conditions such as AD and brain cancer (Association, 2013; Patra et al., 2018). Nanomedicines are comprised of various nanocarriers containing different drugs. The potential for nanomaterials to manage the pathologies of AD is receiving extensive investigation. The treatment of AD currently uses nanostructure-based delivery systems, which will be discussed in the following sections. Most of them are classified as either metallic NPs, organic nanostructures, or lipid-based nanoparticles.

### Liposomes

The phospholipid bilayer of liposomes is the most probable solution to the problem of transporting medications across the BBB. However, it is forbidden to cross the BBB. Numerous surface modifications have been implemented to boost liposomal carrier transport across the BBB (Spuch and Navarro, 2011). Numerous proteins, peptides, antibodies, and other ligand receptors may be present on the surface of the BBB. Transcytosis can be facilitated by applying surface-active ligands, including those found in these compounds. Transcytosis and cationic liposome absorption into the BBB take place simultaneously. Liposomes are typically coated with nutrients like glucose to make it easier for them to move through the body. Once the liposomes have entered the brain, the passive diffusion mechanism can proceed. This process is triggered by the brain’s passive efflux (Noble et al., 2014). Through associated receptors on BBB cells, curcumin-loaded liposomes can substantially improve drug delivery to the CNS (Lajoie and Shusta, 2015). The liposome carrier system that has been modified with a surface with mannose ligand and cell-penetrating peptides (CPPs) has been employed to deliver apolipoprotein E (ApoE2) in the brain injured by AD. The findings show that functionalized liposomes can deliver a significant concentration of genes to the target tissues safely and effectively for the treatment of AD (Arora et al., 2020). Osthole (Ost) is an anti-AD compound because of its prophylactic impacts on hippocampus neurons and anti-Aβ characteristics. Bioavailability and exposure to target sites in the AD mice brain have been addressed by developing an Ost-liposomes carrier system (Kong et al., 2020).

### Polymeric-based nanoparticles

Designing and testing polymeric biodegradable NPs functionalized with antibody and polyethylene glycol (PEG) in transgenic AD mice was productive. According to recent research, exposure to PEGylated NPs can rectify memory deficits and significantly lower levels of Aβ-soluble peptides. The AD disease can therefore be treated with the designed formulation (Carradori et al., 2018). Research has been carried out in which biodegradable polymeric NPs are synthesized using the double emulsion method. The purpose of this study is to determine whether or not loading memantine into these NPs will increase its effectiveness in treating AD. Memantine-loaded NPs can significantly reduce Aβ plaques and AD-related inflammatory processes when used to treat AD brain tissue (Sánchez-López et al., 2018). When applied to mice with AD, targeting the brain with zinc-loaded polymeric NPs can reduce the size of amyloid plaques and help alleviate other neuronal deficiencies (Vilella et al., 2018). To transport the acetylcholinesterase inhibitor known as huperzine A, mucoadhesive and target poly lactic-co-glycolic acid nanoparticles (PLGA-NPs) that have had their surfaces changed with lactoferrin-conjugated N-trimethylated chitosan have been used. The formulation has shown promising results in both its sustained-release action and its ability to target AD pathology (Meng et al., 2018).

Thymoquinone (TQ), a bioactive component found in *Nigella sativa* seed essential oil, has been demonstrated to have a variety of medicinal applications (Javidi et al., 2016). Numerous preliminary pharmacological investigations have been carried out to study the therapeutic utilization of TQ; however, further research is required to decide whether or not it is beneficial in treating neurological diseases. Recent studies have demonstrated that TQ is a potential treatment for AD (Abulfadl et al., 2018).

TQ-containing NPs coated with polysorbate-80 (P-80) may be a viable and dependable method of delivering nanoscale to the brain through the BBB (Yusuf et al., 2021). PLGA is a biodegradable polymer because it can be hydrolyzed into its non-toxic endogenous metabolites, including glycolic acid and lactic acid. Hydrophobic PLGA tends to opsonize and be eliminated by the reticuloendothelial system (RES), despite its widespread use for CNS-targeted drug administration. Since the PLGA-NPs are coated with surfactant P-80, non-toxic, non-ionic, biodegradable, and hydrophilic, they are protected from being opsonized and cleared (Sempf et al., 2013).

**FIGURE 2:**
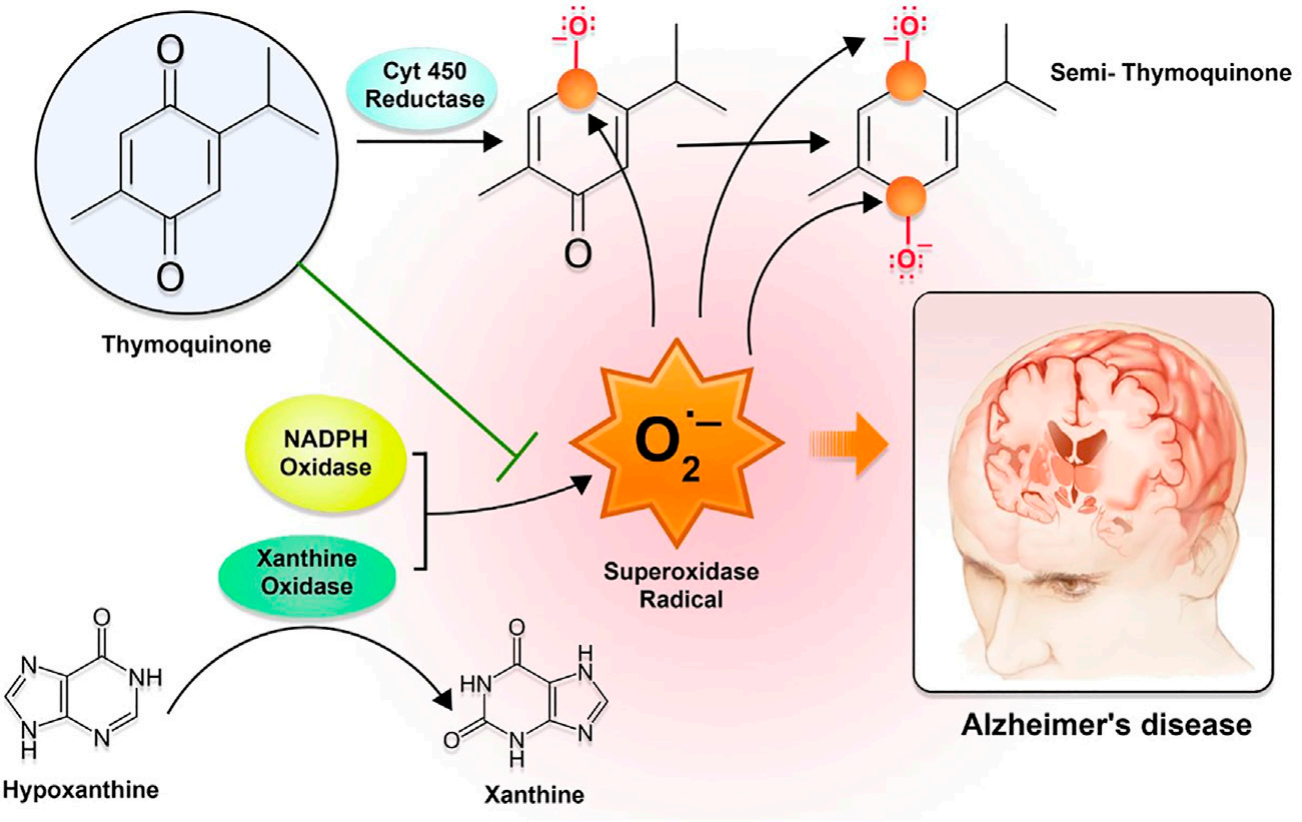
The mechanism of action of thymoquinone with regard to oxidative stress-mediated AD symptoms. The interaction between TQ and PLGA may have led to a further sluggish release, which may have restricted the amount of TQ that was released. The TQ primarily exerts its effect by inhibiting the enzyme xanthine-oxidase and lowering the production of superoxide radicals. In contrast, the semi-TQ that is produced by cytochrome 450 reductase offers electron-deficient platforms at the first and fourth positions, which in turn offers electron-deficient centers for superoxide radicals.

The significant contribution of drug release from the matrix through diffusion caused by pore formation comes from the autocatalytic hydrolytic breakdown of PLGA to lactic and glycolic acid. Through the autocatalytic breakdown of the matrix, it may be possible to accomplish both high porosity and strong drug diffusion. Because of the hydrophilicity of the P-80 coating, TQ from P-80-TQN could be easily derived (Tığlı Aydın et al., 2016; Zeng et al., 2020). The interaction between TQ and PLGA may have led to an additional delayed discharge, which may have restricted the amount of TQ delivery. The primary mechanism by which TQ exerts its effect is by inhibiting the enzyme xanthine-oxidase, thereby lowering the production of superoxide radicals. In contrast, the semi-TQ produced by cytochrome 450 reductase offers electron-deficient platforms at the first and fourth positions, thereby supplying electron-deficient centers for superoxide radicals. Because of the reduction in OS and AD, these processes can bring the number of superoxide radicals in the environment down to a safer level (Figure 2).

The cationic mucoadhesive polymer chitosan (CH) was also used in the study because it can form gels by absorbing water, extending the period at the site of action. When medications are taken orally, CH is said to enhance medication penetration through the nasal mucosa and assist in opening tight junctions, as stated in several studies (Ariful Islam et al., 2015; Wang et al., 2018; Duan et al., 2022). Utilizing this material as a shell makes it possible to employ CH to increase the residence period and improve medication permeation.

Accumulation of inhibitors of Aβ could help treat AD. Various drugs can destabilize the A fibrils *in vitro*, preventing Aβ accumulation and neurotoxic effects. Much research has been done on the inhibitory action of NPs. The functional NPs have the potential to avoid the aggregation of proteins efficiently. Light-activated gold nanoparticles (AuNPs) containing peptides have the potential to cause the disintegration of preformed fibrils. Detrimental ions are precluded from being released from the nanocarrier whenever the surface is appropriately functionalized (Meenambal and Bharath, 2020).

NPs may disrupt small fibers and avoid their accumulation. Experiments may successfully avoid Aβ accumulation and fibrillation when applying the small AuNPs because they decelerate the nucleation mechanism. Theoretical perspectives for therapeutic candidates to treat AD may thus be gained through synthesizing AuNPs.

Curcumin is an antioxidant with low toxicity and a free radical scavenger, a naturally occurring phytochemical (Azizi et al., 2018; Sharifi-Rad et al., 2020). The most appealing method for treating AD is administering curcumin to the brain, inhibiting tau protein accumulation and exhibiting anti-amyloid characteristics at concentrations in the micromolar range (Yang et al., 2022a). Poor stability and bioavailability of curcumin during brain delivery contribute to its reduced brain absorption (Gao et al., 2020). The use of nanocarriers is favored as a means of addressing these concerns because of the safety benefits associated with this method and the increased and sustained brain exposure it provides. It is also possible to establish a stable and sustained distribution of curcumin across the BBB by utilizing a nanoemulsion of red blood cell membrane-coated PLGA particles loaded with T807 molecules implanted on the surface of the red blood cell membrane (T807/RPCNP). The mutual actions of these proteins show strong inhibitory effects of T807, 807/RPCNP on tau-associated pathogenesis (Gao et al., 2020). Additionally, to expedite the phagocytosis of the Aβ peptide and enhance drug permeation, curcumin loaded with chitosan and bovine serum albumin NPs is used to mitigate AD manifestations (Yang et al., 2018). Through various kinase pathways, curcuminoids control the proliferation of neuronal stem cells in the Aβ-induced rat model (Ling et al., 2021). In order to alleviate the symptoms of AD, curcumin that has been loaded with chitosan and bovine serum albumin NPs has been shown to boost the drug penetration and speed the phagocytosis of the Aβ peptide. Other biological benefits of curcumin-based nanomedicines for treating brain disorders include their ability to protect neurons against dopaminergic toxicity by activating the transcription factor Nrf2, which is renowned as a master regulator of the antioxidant response (Szwed and Miłowska, 2012; Yang et al., 2018; Chen et al., 2021).

### Nanogels

It has been shown that the use of nanogels for the delivery of pharmaceuticals is more effective than the administration of free drugs. This was established in comparison to the effectiveness of the administration of free drugs. This happens as a result of a number of reasons, some of which include enhanced cellular absorption of the medication, decreased drug toxicity, higher drug loading, and controlled release of the loaded drug at the targeted site (Neamtu et al., 2017). The potential of nanogels to bind active compounds, macromolecules, and drugs make them attractive drug delivery systems that have been used to address many problems related to various pathologies, such as AD (Aderibigbe and Naki, 2018). According to a recent study, one of the most effective treatments for AD involves delivering deferoxamine as nanogels employing the chitosan and tripolyphosphate approach (Ashrafi et al., 2020). Polysaccharide pullulan backbones modified with cholesterol moieties serve as artificial chaperones that have been proven to alleviate AD pathology by preventing the development of Aβ amyloids (Ikeda et al., 2006). The nose-to-brain delivery of insulin, a candidate drug for AD, has been assessed and found to be increased by employing nanogels as a carrier during a preclinical experiment conducted on mice (Picone et al., 2018). When combined with polysaccharides, the NPs had a number of benefits, including the fact that they were non-toxic, very stable, hydrophilic, and biodegradable (Meng et al., 2018).

### Dendrimers

For the treatment of AD, dendrimers are viewed as a potentially useful compound (Aliev et al., 2019). Combining low-generation dendrimers and lactoferrin to deliver memantine to specific brain regions in AD-induced mice has resulted in a discovery. An important effect on target mice’s memory aspects was noted in recent research (Gothwal et al., 2019).

**FIGURE 3:**
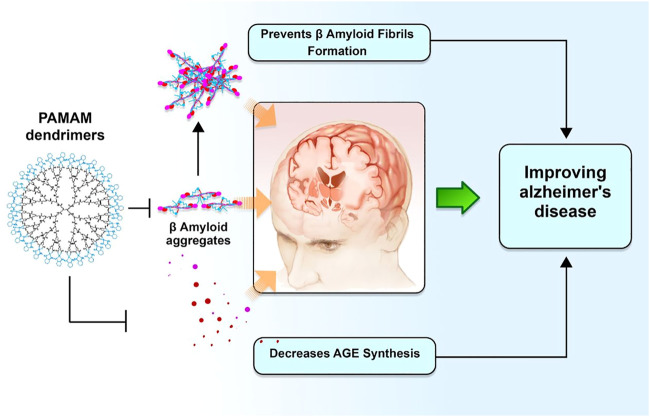
Dendrimers and their potential role in improving AD pathogenesis. Polyamidoamine dimers, also known as PAMAM, serve as modulators for amyloid fibrillar production. This, in turn, enhances biocompatibility and degradation of synapses against A oligomers, hence preventing a deterioration in memory associated with Alzheimer’s disease.

Dendrimers with an ethylenediamine core, generation 4.0 and 4.5, are commonly used to improve the drug solubility and bioavailability for greater permeation across the BBB to target the damaged parts in the brain to increase the efficacy of drug-related CNS disorders such as AD and polyamidoamine (PAMAM). This is done to increase the efficacy of drug-related CNS disorders such as AD and PAMAM (Igartúa et al., 2018; Yang et al., 2022b). Dendrimers composed of a poly (propylene imine) core and a maltose-histidine shell (G4HisMal) have been effectively developed, and they may show significant alleviation of AD manifestations like memory dysfunction. To improve biocompatibility and lessen the toxic effects of medications employed to treat AD, tacrine has also been administered in combination with generation 4.0 and PAMAM dendrimers as nanocomposites (Jin and Wang, 2016; Igartúa et al., 2020). Moreover, the disposable immune platforms for the concurrent identification of the biomarkers for AD have been designed using the nanocomposites of PAMAM dendrimers and gold NPs (Figure 3) (Serafín et al., 2021).

### Micelle

In the form of drinking water, double transgenic AD mice are given a micelle water-soluble formulation of coenzyme Q10 (UbisolQ10). The findings indicate that it is effective in enhancing long-term memories and lowering the concentrations of circulated Aβ plaques (Muthukumaran et al., 2018). The availability and effectiveness of curcumin in treating AD symptoms have been found to increase when Tween-80 is mixed with micelles to develop curcumin micelles (Hagl et al., 2015). In recent research, neuronal N2 cells have examined how PEG ceramide nanomicelles influence the cells. Nanomicelles have been demonstrated to be an efficient tool for mediating tau protein disintegration and inducing autophagy in target cells (Gao and Jiang, 2006). Another study demonstrates the use of curcumin-loaded polymeric nanomicelles as a targeted therapeutic delivery system in conjunction with the glycation method of bovine serum albumin in the presence of phosphate-buffered saline results in significant inhibition of the amyloidogenesis process in mice with AD (Mirzaie et al., 2019). In addition to this, it was demonstrated that an artificial chaperone comprised of mixed-shell polymeric micelles (MSPMs) has been developed with variable surface characteristics that serves as a suppressor of AD. This artificial chaperone was inspired by a natural molecular chaperone. MSPM-based chaperones have the ability to maintain A homeostasis through a variety of mechanisms, including the inhibition of A fibrillation, the facilitation of A aggregate clearance, and the reduction of A-mediated neurotoxicity at the same time (Huang et al., 2014). MSPMs were able to lessen the load of A, dampen the inflammation caused by A, and ultimately repair the cognitive abnormalities shown in APP/PS1 transgenic AD mice (Yang et al., 2019). An additional nanochaperone was developed by designing a VQIINK hexapeptide that was obtained from the tau protein onto the surface of a self-assembly micelle that was outfitted with chaperone-like hydrophobic microdomains and restricted spaces. This nanochaperone is able to collect pathogenic tau without affecting normal tau, and it inhibits their ability to aggregate by a significant amount because to the synergistic action of tau-recognizing peptides and limited hydrophobic microdomains on the surface (Xu et al., 2022).

### Selenium nanoparticles

As was mentioned earlier, one of the most important steps in treating AD is lowering the level of ROS in the brain. Active ROS inhibitors can be found in many different trace elements, including selenium (II), sodium selenite (VI), and sodium selenite (IV). Because selenium and selenite are essential micronutrients for the human body and have the potential for use in biomedical applications of selenium nanoformulation, nanoparticles containing selenium and selenite have been shown to reduce oxidative stress and prevent the cytotoxicity of cells. As a result, they can potentially be used in the treatment of neurodegenerative disorders such as AD (Rajeshkumar et al., 2019). The BBB has been discovered to be permeable to modified selenium NPs containing sialic acid, and their exposure has been shown to inhibit the Aβ accumulation reactions (Yin et al., 2015). Aβ aggregation might also be blocked by using sialic acid-modified selenium NPs coated with high BBB permeability peptide-B6 and epigallocatechin-3-gallate (EGCG) (Zhang et al., 2020). One promising delivery system for AD treatment is a new modified nanoformulation of selenium NPs entrapped in PLGA nanospheres with curcumin, demonstrating potent inhibitory impacts against Aβ accumulation in a transgenic AD mouse model (Huo et al., 2019).

### Antibody-based nanoparticles

When immunotherapy doses are given to treat AD, complications such as meningoencephalitis could emerge (Hoskin et al., 2019). Applying NPs coated with antibodies directed against specific target proteins is the most effective alternative to immunotherapy for locating and dissolving protein aggregates in brain cells. Secondary ion mass spectrometry is employed to image proteins associated with AD in the brain using antibodies coated with metal oxide NPs (Moon et al., 2020). Nanovehicles coated with chitosan and Aβ fragments have been used to target amyloid-containing cells in AD. NP-Aβ absorption across the BBB is boosted by contrast agents like fluorescein isothiocyanate **(**FITC) and Alexa Fluor (Agyare et al., 2008). The therapy of AD with the class A receptor activator XD4 (W20/XD4-SPIONs) and the A oligomer-specific scFv-AbW20 coupled to superparamagnetic iron oxide NPs (SPIONs) has shown some encouraging results (Liu et al., 2020a). Outstanding early diagnostic potential for AD was found in superparamagnetic iron oxide NPs when they were associated with an A-oligomer-specific antibody and a category A scavenger receptor activator (Liu et al., 2020b).

### Receptor-based targeted treatment of Alzheimer’s disease

Through a process known as receptor-mediated transcytosis, macromolecular ligands such as proteins (plasma proteins), hormones, enzymes, and growth factors are transported to the brain. This process begins with the ligand binding to a specific trans-membrane receptor; next, the membrane invaginates, and the receptor-ligand complex forms a vesicle that is transported across the endothelial barrier; finally, the vesicle dissociates In the case of drug carriers, targeting ligands are first linked to the surface of the carrier so that they may bind to the receptors, and only after this step can the medication be released into the intended target region. For the treatment of AD, researchers have investigated a number of receptors, including transferrin lactoferrin, insulin, low density lipoproteins, and toll-like receptors, in the hopes of delivering the drug moiety to the target region in the brain while avoiding the BBB (Wong et al., 2019).

The brain’s capillary endothelial cells contain low-density lipoprotein (LDL) receptors and LDL receptor related protein receptor 1 (LRP1), which may accept or bind a variety of ligands for signaling and scavenging. They are therefore appropriate for the targeted delivery of drugs to the brain. LRP1 is likewise an APOE and APP receptor (Holtzman et al., 2012).

Lactoferrin is an iron-binding glycoprotein and a member of the transferrin family. Both neurons and brain endothelial cells have been found to have high levels of lactoferrin receptor expression. Therefore, lactoferrin receptors may be targeted by employing lactoferrin ligands to deliver medications to the brain in order to treat AD. Transferrin receptors are found in high concentrations in the endothelium of brain capillaries. It is possible to circumvent the BBB when it comes to the delivery of medications for AD by conjugating the drug delivery system with ligands that target the transferrin receptor. In a similar manner, substantial amounts of insulin receptors have been discovered on the cell surface of brain vascular endothelial cells. When compared to transferrin receptors, insulin receptors are substantially more successful in crossing the BBB, which means that targeting insulin receptors could more effectively carry therapeutic molecules into the brain (Wong et al., 2019).

### Combinatorial nanomedicines in the management of Alzheimer’s disease

In view of the failure of treatments aimed at a single target, it would seem that multi-target combination therapies, which include the simultaneous administration of a number of drugs, offer the most potential for treating AD. It has been shown that nanomaterials are capable of delivering many drugs (such as chemical compounds, genes, peptides, and antibodies) all at once, suggesting that they may have a future use in the treatment of AD. There have been some really ground-breaking advances made in the treatment of AD as well as in its diagnosis because to nanomaterials (Chopra et al., 2022).

For instance, in the Chinese patent CN110559454B (2022), CRT (cathode ray tube) targeting peptides and QSH (quadrupole superparamagnetic ferrite) are used to modify a medicine-carrying nano micelle. This nano micelle is used to transport a medication that contains an anti-amyloid protein and superparamagnetic ferrite, and it is intended to treat AD. The nano composite medicine incorporates both the diagnosis and treatment of AD as well as image tracing using MRI technology. The invention combines a QSH targeting peptide and a CRT targeting peptide in order to enable drug-loaded nano-micelles to pass across the BBB by targeting both AD protein and transferrin. This is accomplished by combining the QSH targeting peptide and the CRT targeting peptide. By concurrently focusing on AD protein and transferrin, it is possible to boost the concentration of a medicine at a specified spot while also extending the amount of time it takes for the drug to have its effect (Patents, 2022a).

The invention protected by patent CN110507830A centers on a particular kind of nano-probe and the process of producing it for the purpose of detecting AD pathogenic protein (CN110507830A, 2022). In order to create the multi-modal nano-probe that is the subject of the present invention, a polyethyleneglycol derivative and a phenothiazine derivative are used as key building blocks. An extra-small ferrite nanometer particle is located in the center of the nano-probe, and the nano-outside probe’s layer is composed of a polyethylene glycol segment that is coupled with the phenothiazine derivative. In addition to its one-of-a-kind impact of improving the contrast of near-infrared fluorescent labels and its effect of improving the contrast of T1–T2 nuclear magnetic resonance images, the multi-modal nano-probe may be particularly useful when used in conjunction with beta-amyloid protein patches. The probe has a high application potential in the early detection of AD, in addition to its small size, great biocompatibility, radiation-free features, and a range of without any neurotoxicity (Patents, 2022b).

## Gene therapy

Recently, gene therapy for AD has received a lot of interest. A gene that expresses an enzyme or growth factor was included into the medication as a generic option. The fundamental objective of this strategy is to maintain the therapeutic expression levels of chosen genes over the long term. Altering or activating certain proteins that are involved in the pathological process of neurodegenerative illness is one way to achieve neuroprotection and neurorestoration. These two goals can be accomplished simultaneously. When it comes to the treatment of neurodegenerative diseases, gene therapy is an exceedingly complex process that involves a number of variables, including the specificity of time and place, the regulation of genes, the transport of genes, and more (Sudhakar and Richardson, 2019). The target illness dictates whether an integrating or non-integrating form of gene transfer is used, and whether *in vivo* or *ex vivo* therapy is administered for treatment (genetic disease vs. complex acquired ailment). To a large extent, this is achieved by the enhancement of the gene, the inhibition of the gene, and editing of the genome (Soofiyani et al., 2013). Additionally, there are small, single-stranded antisense oligonucleotides that connect with RNA messengers to prevent a particular gene from being translated (also known as AS-Ons). The antisense oligonucleotides known as IONIS MAPTRx, which are intended to restrict the synthesis of tau, have been in clinical studies with the intention of serving as a novel technique to reduce tau production in the brain, as has been known for some time (Jadhav et al., 2019).

## Conclusion and future prospective

Improving the delivery of drugs, therapeutic proteins, and antiamyloids across the BBB is one of the applications of nanotechnology that can be found in treating AD. Bioimaging and proteomics advancements are potential uses of nanotechnology in treating AD. The potential for chronic toxicity needs to be further investigated for potential clinical applications, notwithstanding the recent developments in using nanotechnology for AD treatment. The future of nanomedicines utilized in AD seems bright. We recommend that the existing procedures be revised in order to take into account the aspects that have been ignored at the nano–bio interface. This will help to reduce the likelihood that the results will be misinterpreted in the future. It is also advised that multifunctional NPs with many therapeutic capabilities be used (for example, providing a range of therapeutic moieties to regulate inflammation, oxidative stress tau phosphorylation, and mitochondrial dysfunctionality). Furthermore, the difficulties of producing reproducible NPs on a big scale must be addressed. Given the present medications’ major targets of tau proteins, neuroinflammation, and Aβ proteins, there is an urgent need to create treatments with novel targets that may not only cure symptoms but also prevent disease development at an early stage, eventually leading to a higher quality of life.
